# Factors Impacting Pharmaceutical Prices and Affordability: Narrative Review

**DOI:** 10.3390/pharmacy9010001

**Published:** 2020-12-23

**Authors:** Kah Seng Lee, Yaman Walid Kassab, Nur Akmar Taha, Zainol Akbar Zainal

**Affiliations:** Faculty of Pharmacy, University of Cyberjaya, Cyberjaya 63000, Selangor, Malaysia; dryamankassab@yahoo.com (Y.W.K.); akmar@cyberjaya.edu.my (N.A.T.)

**Keywords:** mark-up mechanism, pharmaceuticals pricing, availability, pharmacoeconomics

## Abstract

Increasing prescription drug pricing often reflects additional work stress on medical professionals because they function as financial advisors for patients and help them manage out-of-pocket expenses. Providers or prescribers wish to help patients with prescription costs but often lack related information. Healthcare plan providers try to display prescription and drug cost information on their websites, but such data may not be linked to electronic prescription software. A mark-up is defined as the additional charges and costs that are applied to the price of a product for the purpose of covering overhead costs, distribution charges, and profit. Therefore, the policies implemented in the pharmaceutical distribution chain might include the regulation of wholesale and retails mark-ups and pharmaceutical remuneration. If mark-ups are regulated, countries are highly recommended to use regressive mark-ups rather than fixed percentage mark-ups. This narrative review provides insights into the framework of pharmaceutical mark-up systems by describing different factors impacting pharmaceutical prices and affordability. These include the interplay of medicine pricing and the supply chain, the impact of pertinent laws and regulation and out-of-pocket expenditure.

## 1. Introduction

Countries across the world are implementing various pharmaceutical pricing policies and procedures in order to cope with increasing drug prices [1]. Most countries implement a combination mechanism to determine and regulate pharmaceutical product prices. Four policies and strategies that are most commonly used are the regulation of mark-ups and distribution chains, external/international reference pricing, promotion of generic medicine use, and tariff/tax exemptions. Among the countries that implement regulation on mark-up distribution are Malaysia [2], Indonesia, Myanmar, Laos, Vietnam and the Philippines, but only in the public sector, so the private sector is left unregulated. Only Malaysia [3], Vietnam and Laos practice external reference pricing to regulate the price of pharmaceutical products while most of the ASEAN countries, especially Malaysia and Indonesia, apply generic medicine promotion to enhance the use of generic medicines as they are much cheaper [4]. However, the availability of generic substitutions in each country varies greatly.

A mark-up is defined as the additional charges and costs that are applied to the price of a product for the purpose of covering overhead costs, distribution charges, and profit [5,6]. Therefore, the implementation of pricing policies in the pharmaceutical distribution chain might include the regulation of wholesale and retail mark-ups and pharmaceutical remuneration. The manufacturer’s selling price is the price of a product defined by its producer, and this is also the initial price of the whole supply chain. Distribution costs and the wholesaler’s mark-up are the overhead costs applied on top of the manufacturer’s selling price. Following that, retailers add their own expenses to cover procurement and marketing costs. The add-on price is then known as the retailer’s mark-up. The reason for putting mark-ups at various levels is to measure the supply and demand of a product in the market. When the price is higher, producers are motivated to produce more, hence boosting product sales. However, unchecked mark-ups could bring the flow of supply to a standstill if the majority of end-users cannot afford to buy the products. If mark-ups are regulated, countries are highly recommended to use regressive mark-ups rather than fixed percentage mark-ups [7,8]. Regressive mark-ups mean lowering the mark-ups for higher-priced products. The fixed percentage mark-ups are less favourable, as they might contribute to a higher net margin for the higher-priced products and increase the price of low-cost drugs. The procedures for calculating and regulating the size of mark-ups range from 0% mark-up allowed until more than 100% mark-ups. This proves that a single model does not suit all countries and must be modified according to the setting. With the implementation of regressive mark-ups policy, many countries found it beneficial to stop excessive charges being added to medicines as they pass along the supply chain. However, this policy requires a strong strategy of enforcement by the government as well as high-level political support to make sure it is efficient to control the drug price [6,9,10]. This narrative review provides insights into the framework of pharmaceutical mark-up systems by describing different factors impacting pharmaceutical prices and affordability. Price components, supply chain hierarchy, and regulatory measures affecting drug pricing will be discussed in this review.

Pharmaceutical price control measures are the mechanism used by low- and middle-income countries to keep drug prices in check while increasing affordability. In fact, analysis shows that even with the same policy, market differences between low-, middle-, and high-income countries can lead to significantly different results. According to economic theory, if the marginal cost does not change, the market reaction to price cap adjustments is an increase in supply. The cost of supply chains to rural areas is particularly high due to the lack of densely populated towns and cities, paved roads and other necessary infrastructure. However, increasing marginal costs can obscure the market’s reaction to price cap adjustments and reduce market supply. The impact is especially severe if the law significantly reduces market-level supply, as in this case companies are most likely to withdraw their products from rural areas, and so health services become unavailable in these areas. Nevertheless, every policy has advantages and disadvantages once it has been implemented. The regulation of mark-ups in the absence of any price control strategy causes medicine prices to drop. Plus, the implementation of this policy is less complicated as compared to the other available options because it only needs minimal information about the price of goods and the supply chain along with some enforcement capacity [11]. On the other hand, this policy may have a negative effect on availability and access through price fluctuation. Other than that, there would be a risk of higher prices if the development of the mark-up structure lacked transparency.

## 2. Interplay of Medicine Pricing and Supply Chain Mechanism

### 2.1. Pharmaceutical Price Components

The final selling price of a drug is made up of several price components, one of them being the manufacturer’s selling price (MSP). MSP comprises freight costs, tariffs and taxes by the local government, overhead costs, procurement costs, and other expenses. These processing fees are significant; in certain cases, they can even exceed 100% of the production cost of a drug. Other than MSP, each level in the pharmaceutical supply chain has its own costs which ultimately accumulate on the price of the medicine. In this context, each and every price component overlaps on the base (MSP) on which the subsequent costs are levied. When a small price component is imposed, its effect is multiplied across the supply chain, contributing to a significant price increment. Therefore, it is crucial for governments, non-government organisations (NGOs), and social insurance scheme providers to revise a plan to regulate these price components. Actions should be taken to reduce the selling price of medicines, increase the efficiency of medicine distribution systems, and ensure the reliability of international price comparisons if external reference pricing is used.

As every country has its own pharmaceutical policies, the price components vary as well. In some countries, policies that restrict price mark-ups on certain types of drugs are in place to ensure affordability. Others might introduce tax exemptions or tariff discounts, or distribute originator brand and generic drugs through different channels. In general, a pharmaceutical supply chain could consist of the following price components:

(a) MSP
(b) Insurance and freight
(c) Port and inspection charges
(d) Pharmaceutical import duties
(e) Mark-ups by importers, wholesalers and retail distributors
(f) Value Added Tax (VAT)/Goods and Services Tax (GST)
(g) Dispensing fees

Five stages in a pharmaceutical supply chain can be identified in order to clarify the impact of price components:

Stage 1 focuses on MSP, which includes insurance and freight costs. For locally produced medicines, Stage 1 cost is the MSP for the recommended or surveyed pack size, coupled with the logistic costs when the medicine is transported using domestic transport to the procurement unit. For imported medicines, the Stage 1 cost is the MSP plus international cost, insurance, and freight (CIF).

Stage 2 discusses the landed price, which is the sum of all other price components charged on procurement and delivery. The landed price comprises banking fees for foreign currency transactions, inspection costs (either pre-or post-shipment), port fees (docking, storage, handling, in-port insurance), customs clearing, import tariffs and importer’s mark-up. Any fees collected by the central authorities are included in the landed price. The landed price also includes logistic costs such as local transport charges to the purchasing warehouse, the importer or the wholesaler. However, when the stock leaves the purchasing warehouse, the landed price does not take domestic storage and distribution costs into account.

Stage 3 involves the wholesale price, which is compounded on the landed price. The additional costs come from the wholesaler’s overheads such as quality control fees, storage and warehousing costs, handling costs, profit margins, and distribution costs to retailers. Many of the price components might already be included in the wholesale mark-up, so they should not be counted twice.

Stage 4 highlights the retail price. The retailers’ (hospitals, GP clinics, pharmacies) selling price is based on the wholesale selling price, added with the retailers’ expenses: storage, handling, overhead expenses and profit margin. Similarly, care should be taken so that these price components are not duplicated.

Stage 5 is the dispensed price. The dispensed drug price is the sum of Stage 4 price and dispensing fees and taxes (VAT or GST), whichever are applicable. Where there is no dispensing fee or sales tax, Stage 5 costs will be voided and the Stage 4 price will be the final selling price [12].

### 2.2. Pharmaceutical Supply Chain

A typical pharmaceutical supply chain is formed by one or more of the following:

(a) Primary manufacturing (possibly including contractor sites);
(b) Secondary manufacturing (possibly including contractor sites);
(c) Market warehouses/distribution centres;
(d) Wholesalers; and
(e) Retailers/hospitals.

The majority of the constitution of a pharmaceutical supply chain can be studied through three main processes: manufacturing, distribution, and retailing [13].

Manufacturing of the medicine: The supply chain begins at the manufacturing stage. Before a pharmaceutical company is allowed to produce a drug, sufficient research and development work has to be performed prior to regulatory approval. After going through a series of manufacturing and quality assurance processes, a medicine is eventually ready to be commercialised and released into the market. Each type of drug has to comply with specific steps and requirements, which differ according to country, before it can be launched into production.

Distribution to the dispensing point: The second process involves the transportation and handling of the medicine from the manufacturer to the retailer. A retailer can be a hospital dispensary, retail pharmacy, hospital or dispensing doctor. In some cases, the ministry of health (MOH) can be the sole retailer of drugs in the whole country. The distribution of drugs can be complicated as it largely depends on the manufacturing company’s location, the urgency of the drugs, handling requirements, and most importantly the location of the retailer since the difficulty to access the area can vary between cities and more rural areas.

Dispensing to the end-user: After the drugs are transported to the retailer, a retailer has the responsibility to dispense the proper dosage and form of drugs to the end-users. The act of providing medicine supply is the final step of a pharmaceutical supply chain. In addition to dispensing drugs, this final step also involves after-sales services such as providing the correct information to the end-users and processing reimbursement claims, so as to ensure the end-users fully benefit from the product they purchased.

### 2.3. Private Sector Supply Chain

**Figure 1:**
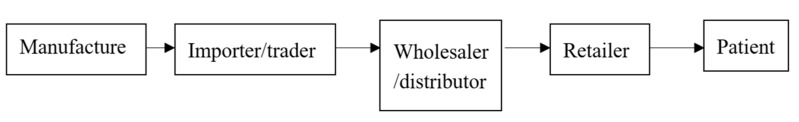
Traditional supply chain for medicines in the private sector.

The traditional private sector pharmaceutical supply chain is illustrated in Figure 1, whereby the supply chain starts off with the manufacturer or importer of the drug, who is responsible for producing the drug locally, or importing the drug from other countries. The drug will later be sold in bulk to a wholesaler, who also functions as a distributor. A wholesaler sources a wide range of drugs from multiple manufacturers/suppliers. In cases where the drugs are originated from other countries, a wholesaler sometimes conducts the drug imports themselves. The next step of the supply chain is followed by retailers who obtain the drugs from wholesalers in smaller quantities. Retailers can be private hospitals and clinics, private pharmacies, or other government-approved sellers.

**Figure 2:**
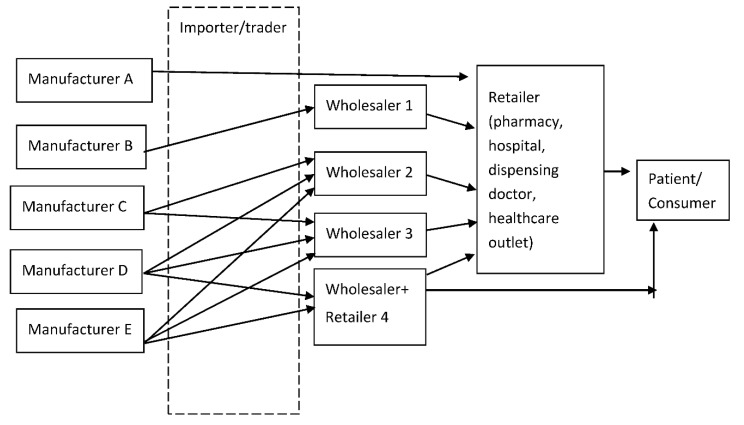
Pharmaceutical supply chain models in private sector.

The private sector supply chain functions the same way as the general pharmaceutical supply chain. However, to meet current market conditions and marketing strategies, many countries have taken the initiative to modify the traditional distribution model. The alternative supply chain model is shown in Figure 2 where only one and/or two may be in operation, depending on the market situation. As shown in Figure 2, manufacturer a sells directly to the retailer and manufacturer B has an exclusive distribution agreement with Wholesaler 1, who is a dedicated short-line wholesaler which carries limited products from manufacturer A. Manufacturer C does not make products available to wholesalers, unlike manufacturers D and E who welcome most wholesalers. Wholesaler 2 may sell on to other wholesalers or on to retailers whereas Wholesaler 4 not only sells to retailers but also directly to patients. To further elaborate this, some manufacturers have made their products exclusive to certain wholesalers or distributors. Likewise, some wholesalers may carry the full range of available stock (full-line or fully-sorted wholesalers) while others may only be able to access certain products or certain manufacturers (short-line wholesalers). Full-line wholesalers may require assistance from primary stockholders and/or short-line wholesalers to make sure they are able to procure and manage the large inventory that full-line wholesalers would have to carry and put into distribution. When facing geographically challenging distribution lines, a series of wholesalers may have to come together in order to supply the products to retailers and end-users.

The mark-up mechanism usually appears in retailer-dispenser and dispenser–end-user relationships. A mark-up fluctuates whenever commercial practices involving discounts and rebates are in place. The presence of price mark-ups creates competition between supplier and buyer weighing on the quantity purchased or sold. To stimulate more purchases by wholesalers or retailers, suppliers often use various marketing mechanisms such as cash rebates, volume discounts, and bundle sales. When the buyer offers to make their sales data available for suppliers’ access, or make early payments, additional discounts may be provided in order to increase the margin of the buyers. However, the discounts could be recovered by the supplier through improved sales, higher cash flow or improved business intelligence. By doing so the supplier also decreases the transparency of the actual selling cost of a product, especially with rebates where the invoiced price does not equal the actual payment that will be made back to the buyer by the seller [14].

Within the healthcare industry, the pharmaceutical supply chain has to be properly managed to ensure the smooth operation of health services. Cost-wise, drug supply is estimated at 25% to 30% of the overall operational costs for hospitals. The primary manufacture is in charge of the active ingredient present in the product. When producing different products, the production line has to go through long downtime in order to perform decontamination procedures. Therefore, mass production is effective to offset the lost time. In secondary production, the manufacturer receives the products (drug tablets and capsules) which have been converted from the active ingredients. In conjunction, the number of product lines can be increased to boost production capacity.

To release the finished products into the market, the distribution method could vary depending on the market demand. The dominant intermediary (middleman) is called the wholesaler. In the United Kingdom, approximately 80% of volume supplied by the manufacturers flows through this channel. When there is a large demand, such as from hospitals and retailers, shipments could be received directly from the manufacturer distribution centres. Group purchasing organisations could help consolidate the requisition of drug supplies for hospitals.

## 3. Pricing Mechanism: Legislative Context

### 3.1. Private Healthcare Facilities and Services Act/Regulations

The Private Hospital Act 1971 was implemented in Malaysia to control the registration, licensing, and inspection of private healthcare centres, including private hospitals, nursing homes, and maternity centres. Operating licenses ensure the operations adhere to basic service standards and meet the minimum requirements, and are renewed annually. However, the Private Hospital Act and its associated regulations have grey areas concerning services or facilities other than private hospitals, such as private community GP clinics, private pharmacies, private laboratories, ambulance services, haemodialysis centres and hospices [15].

In Malaysia, the Private Health Care Facilities and Services Act (PHCFSA) and Regulations (PHCFSR) enforce laws in the registration, licensing, and operation of private healthcare facilities. The aim is to promote better service and protect the wellbeing of patients. However, the laws are seen to be overly strict and criminalizing the private sector. Private healthcare operators are upset with the enforcement of administrative micro-management, unreasonable heavy fines and restrictions, and the unfair nature of the regulations, inspections and implementation [16].

### 3.2. Tariffs/Taxes Exemption

There are two major types of tax, direct tax and indirect tax. Direct taxes are levied by the government on the income of individuals and corporations, while indirect taxes are imposed on the price of goods and services [17]. WHO/HAI surveys on medicines’ affordability and availability states that taxes are the third-largest part after the manufacturers’ price and distribution mark-ups. The mentioned additional costs would lead to a higher total cost paid by patients [18]. According to WHO, the selected low-income countries that applied high tariffs caused an increase of the price of medicinal ingredients by 23 percent and the price of the finished product by over 12% [19]. WHO Guideline on Country Pharmaceutical Pricing Policies published in 2018 also identified the reduction or exemption of taxes on medicine, especially sales taxes is one of the main interventions that could increase medication affordability [17]. Similarly, the same WHO/HAI policy review also concerned the indirect taxes on medicines such as value-added taxes (VAT) and sales taxes that are regressive as the percentage price paid by either rich or poor customers is the same. The policy review concluded that VAT on medicines in high-income countries ranges from 0% to 25% while some countries such as Australia, Japan and the Republic of Korea [13] exempted medicines from these taxes. The benefit of tax exemptions or reductions for pharmaceutical products is providing an equity impact on the poor but the downside of it is that it causes a loss of revenue for national governments. It also might have a negative impact on some aspects of the health care system [17]. As a guideline, WHO has recommended countries to consider exempting essential medicine from taxation as well as ensuring there are effects of decreasing costs for the customer if the strategy is implemented.

### 3.3. External/International Reference Pricing

According to the WHO Guideline on Country Pharmaceutical Pricing Policies, the external reference pricing (ERP) policy is one of the suggested methods for low- and middle-income countries [20]. ERP is the approach of using the price of a pharmaceutical product in one or several nations to derive a benchmark or reference price in order to set or negotiate the price of the product in a given country [21]. In short, the purpose of ERP is to provide institutional purchasers and price regulators with a target, benchmark or reference price for setting or negotiating the price of a pharmaceutical product [22]. In certain countries, solely ERP is used to determine prices while other countries use ERP in combination. A total of 24 out of 30 OECD countries and 20 out of 24 assessed European countries restrict the use of ERP to on-patent medicine only, while developing countries use it for both on-patent and off-patent drugs.

Although some claims have been made that ERP has been an effective method to decrease medicine prices, there is still no supporting evidence from monitoring reports or rigorous analytical studies [23]. There are several advantages and disadvantages of ERP, sometimes known as international reference pricing (IRP), which is a simple and easy-to-implement method but still needs some modifications, for example, ease of access to price-related information, identifying sample countries and exchange rates that demand certain technical knowledge. ERP is implementable when resources are relatively constrained and it gives fast information to regulators and other policy makers. This is the reason for small countries to apply this approach as they have limited capacity to implement other alternative pricing mechanisms [24]. On the other hand, ERP has major limitations as the price information is not always available, while the available price is usually heterogeneous and difficult to be adjusted to the required type of price [17]. Apart from that, the difficulties faced when implementing this mechanism are the issues when comparing prices in different countries as the strengths, pack sizes and active ingredients might vary among countries, and unit prices are lowest when purchased in bulk [25]. The level in the supply chain in which the prices are being compared also causes further differences between the comparator countries due to margins and taxes [25]. The aspects that are mainly being used to select the referenced countries are the same region, similar or comparable income levels and similar socioeconomic status [14].

## 4. Patient Medicine Spending

### 4.1. Out-of-Pocket Spending

In Malaysia, the Full Paying Patient scheme was introduced in 2007, which allows a patient to receive treatment from a specialist of choice and entitles them to use additional facilities by paying for each treatment or service provided at a public hospital. Part of the charges imposed on the patient is used for medical staff reimbursements. Through the scheme, specialists in the public hospitals can gain a supplementary income, thus preventing the migration of health providers from the public sector to private hospitals, as the private sector tends to offer high pay to attract talents. In short, the Full Paying Patient scheme collects medical fees from each medical treatment process and channels the money to the specialists, while giving certain benefits to the patients such as choice of specialist and shorter waiting times. Although the government has assured that the quality of healthcare for patients would not be biased, and patients who cannot afford the charges under the scheme would not be neglected, the public is still fearing that non-paying patients will be ignored since the attention will be mostly given to those who are at the front of the queue. Therefore, the scheme is seen to be a penalty to the low-income group and a tactic by the government to safeguard its specialists [16].

A typical Malaysian has to bear 75% of his or her medical expenses via out-of-pocket spending [16]. The amount accounts for private prepaid plans such as health insurance, or simply purchase of healthcare services or supplies [26]. In terms of GDP, out-of-pocket expenditures accounted for 1.44% in 2015, which translates to a relatively high 36% of total expenditure. The truth is, out-of-pocket spending has been increasing marginally between 2000 and 2013. Meanwhile, in other OECD countries such as Mexico and Turkey, out-of-pocket expenses have been reduced thanks to the introduction of health financing plans, health budget allocations, or social insurance by the government.

Support of out-of-pocket spending is one of the methods to provide healthcare financial support but it is not a long-term solution. This is because incomparable expenditures are not optimised for bulk purchase of services, requisition discounts, or outcome-linked payments. Moreover, it increases the financial risk for individuals as the cash flow does not contribute to consumption smoothing or redistribution in the community [16].

### 4.2. Out-of-Pocket Costs

Initiatives introduced by governments are critical to ensure healthcare expenditure remains at the estimated range. The financial dynamic dictates that any increase in healthcare expenditure will result in high out-of-pocket (OOP) payments for healthcare services for patients or consumers. A patient’s OOP payment is their expenses for medical care which are not covered under their insurance policy. In the past decade, strategies have been introduced in the US to narrow the Medicare Part D coverage gap in order to reduce the OOP costs for patients taking drugs with exorbitant prices, especially biologics for immunotherapy and cancer. However, yearly increases in list prices and the introduction of newer and more expensive drugs mean there have been limited savings for beneficiaries. One particular study examined the Medicare Formulary and Pricing Files from 2010 to 2019 for the most widely prescribed biologic drug for the treatment of rheumatoid arthritis to estimate the OOP cost/year. The findings showed an average 22% OOP cost increment in 2019 when compared with 2010. It is obvious that patients in the United States might have paid less in 2011 but soon after that, the Medicare listed prices for the biologics saw an upward trend, even though the coverage gap was narrowing during the study period [27]. Similarly, Borrelli and McGladrigan assessed the OOP costs of first-line oral treatment for relapsed/stage IV renal cell carcinoma pazopanib, sunitinib, axitinib and cabozantinib. Their report revealed that, on average, even with the Medicare scheme, the OOP expenses are around USD 9644 to 15,622 for one year’s supply [28].

Healthcare professionals have a role in alleviating patients’ financial burden by being more aware of the costs of medicines that they prescribe. Pharmacists and nurses should also be aware of medication costs as they are involved in medicine prescriptions. For example, the Department of Health in England implemented independent prescribing by nurses and pharmacists in order to improve patients’ access to medicines [29].

A previous study found that California-based physicians are not equipped with knowledge about formularies and OOP costs to help patients better control their expenses on prescription drugs [30]. This is because the physicians have the impression that it is the responsibility of the pharmacist to deal with cost-related issues. However, it is physicians who face patients directly, not the pharmacists. Besides the health professional, the patients themselves play important roles in helping managing OOP money. High OOP payments may leave insufficient income for other necessities as well as negatively impacting low-income patients’ access to medications. Therefore, patients from low-income groups will try to avoid treatments or taking medications, resulting in poor adherence to the medication. This will consequently affect their health status and quality of life (QoL). The awareness of patient OOP costs is limited by the willingness of the patient to discuss the financial situation with the prescriber. If patients are not willing to share their financial problems as they consider it as a private matter, prescribers cannot help them at all. But, if patients are open to discussion on their personal finances, then the aim of ensuring patients get cost-effective medicine will be easier to achieve.

One study found that the trust relationship between the prescriber and patient would enhance the patient’s willingness to discuss health-care costs [31]. Thus, it is crucial for the prescriber to be skilled in communication to gain trust from the patients in order to make patients more receptive to discussing their OOP costs.

## 5. Discussion

Prices of medicines can be determined by multiple factors, but differential pricing is mainly controlled by pharmaceutical companies and the supply chain. To monitor price changes, WHO/HAI adopts a price comparison between countries. The major difficulty in doing this is the selection of the control price to be used as a comparison basis. On the other hand, drug prices can be influenced by buyer strategies. Buyers could analyse pharmaco-economics and then try to negotiate. Governments could also intervene in the pharmaceutical market by imposing price and profit controls on manufacturers, performing reference pricing and brand premiums, using international benchmarking, reducing tariffs and taxes, fixing production margins, and implementing capitation systems. Another noteworthy factor is globalization. Economic globalization has encouraged drug producers to delocalize manufacturing sites [32]. Ever since the advent of globalisation, pharmaceutical prices have been influenced by compulsory licenses for production, manufacturing, and distribution. The parallel importation of drugs is common in the European Union (EU). It could be beneficial to drug traders as a branded medicine can be sold at a cheaper price in one member state and later sold below the normal local price when exported to another country. Big players in the industry are offering price reductions voluntarily, making antiretroviral therapy affordable in low-income countries dealing with the HIV/AIDS pandemic [19].

The advocacy group Public Citizen found that drug companies in the Fortune 500, an annual listing of the top 500 United States publicly listed companies, made a profit of 17 cents for every dollar of revenue and a return on assets of 14.1%. The statistics greatly surpass the overall Fortune 500, which has an average profit margin of 3.1 cents per dollar and a return on assets of 2.3%. A study by Dave et al. found that there is no strong correlation between market competition levels and market sizes when the two aspects are associated with drug pricing. On the contrary, compared to expensive generic drugs, the likelihood of cheaper drugs getting a surge in price is higher. This is logical when demand is higher than supply. The same concept applies to pharmaceuticals; when pharmaceutical companies are not able to produce enough, the product will be sold at a higher price due to limited supply. The results of the study suggest that there is a higher tendency for shortages in generally affordable generic drugs, which could be a consequence of many different causes, but primarily because their profitability is lower than that of expensive drugs. Generally, manufacturers prioritize higher, or if possible, continuous production of products that can bring in higher profits when the situation allows it. In the United States, the frequent occurrence of drug shortages might be due to its correlation with the reimbursement policies by the US government [33]. However, even with the reimbursement policies in place, the drug pricing across the United States is still higher than in other developed nations, for example, European nations [34]. The United States’ Institute for Clinical and Economic Review examined the price change between 2016 and 2018 of the top hundred highest revenue medications in the United States and reported on Humira^®^ (Adalimumab), Rituxan^®^ (Rituximab), Lyrica^®^ (Pregabalin), Genvoya^®^ (Elvitegravir, Cobicistat, Emtricitabine, Tenofovir), Truvada^®^ (Emtricitabine/Tenofovir Disoproxil Fumarate), Neulasta^®^ (Pegfilgrastim), Cialis^®^ (Tadalafil), Tecfidera^®^ (Dimethyl Fumarate) and Revlimid^®^ (Lenalidomide) [35]. The findings indicated that among the top nine medications that recorded the largest price increase, only Genvoya and Revlimid presented new clinical trial findings or new indications that could justify the price hike. Therefore, it is prudent to suggest that the increase in drug prices, as evidenced by the pricing strategies of major pharma companies, is profit-driven [35]. Drug companies take opportunity cost of capital, research and development (R&D) cost, marketing fees and post-approval clinical trials into account, and impose the cost on consumers. The counterargument given by the pharma companies is the extremely high initial research and development investments for drug development. It is a well-known fact that the whole product development process, from drug lead discovery to the patent filing, drug approval and marketing could easily consume USD 1 to 3 billion [36,37]. Therefore, drug companies aim to keep patents in force for as long as possible to prevent a price drop once the patent expires. To do that, drug companies manipulate their products in accordance with the legislation to extend exclusivity, arrange schemes with generic companies, or raise a new patent for an “improved” version of the original product.

Similar to all other economic entities, the pharmaceutical industry has been seriously affected by the pandemic outbreak. The majority of active pharmaceutical ingredients (APIs) for generic drug production is supplied by India and China. Indian manufacturers are highly dependent on China for their drug production. India procures around 70% of its medicine formulations from China, which is the number one global APIs producer and exporter by volume. The outbreak of COVID-19 has once again emphasized the reliance of the Indian pharmaceutical industry on China for APIs [38]. Balfour proposed several strategies to promote APIs production in India: a pharmaceutical regulatory relaxation especially for APIs producers; ensuring reliable supply chain management; providing an exclusive economic zone for domestic pharmaceutical hubs; and considering sustainable financing and tax exemption. While China is gradually picking up pharmaceutical production and exportation, the supply will not be sufficient to support the massive usage of APIs worldwide. Other major APIs exporting countries, India being one of them, are still holding on to exports of APIs and related drugs. The United Kingdom has started to restrict parallel exports so that the stock for its citizens is secured. Other countries, particularly European countries and the United States, are planning to move their production line back to their own region to prevent subsequent losses amid the pandemic outbreak and unprecedented lockdown in exporter countries [38].

In Europe, the drug price fluctuation can be seen from supply-related and demand-related factors. The recent COVID-19 outbreak has had a notable effect on the pharmaceutical supply chain and patients’ treatment accessibility. The ongoing and possibly future APIs/raw material shortage will risk the sustainability of patients’ demands. To counter the shortage effect, quality risk management plans which focus on the prevention and management of drug supplies cut-off should be implemented on manufacturers. Some of the proposals can generate quick results in preventing shortages, such as upscaling of raw material suppliers and increasing the number of available stocks. In reality, not all alternatives are applicable in every scenario due to the high economic impact. European countries are promoting a model that involves the scope of the structure of procedure and decision trees, to visualise risk mitigation plans for the EU. The idea is to cater to the domestic needs of tailored or essential healthcare assistance. At the same time, the EU has advised regulatory authorities and drug supply chain stakeholders to communicate in order to solve the distribution issue of unavailability in remote geographic localities, and ultimately to curb drug shortages. Consequently, a task force team responsible for the resolution of the shortage crisis is much needed at both national and European levels [32].

## 6. Supply Chain and Medicine Pricing

In most pharmaceutical markets, the distribution of drugs is carried out by distributors/importers and wholesalers. They are the intermediaries between manufacturers and retailers to ensure the market can receive a smooth supply of medicine. For imported medicines, extra steps should be taken as the logistics of supplying the drugs into a country are often complicated due to international regulations and the country’s own policies. The complexity associated with the procedures and parties involved differs based on the product itself, market demand, and distribution strategies [13].

Logistical planning is one of the major challenges faced by distributors when supplying a myriad of pharmacies with pharmaceutical products obtained from a large pool of manufacturers within a short time. Distributors have to comply with certain distribution standards to ensure the handling of pharmaceutical products meets the retailers’ requirements, and at the same time to prevent misconduct that would result in potential losses. In other words, a distributor’s functionality is to provide distribution services to its clients by first investing in inventory management. Typically a distributor holds a few months’ worth of inventory which means inventory costs such as warehousing, capital, and potential obsolescence. In cases where the distributor is also a wholesaler, it has to bear additional expenses coming from interest imposed and risk of delayed repayment or, at worst, default. In Kenya, for example, importers often refuse to deal with domestic currency. Therefore, when purchasing products from manufacturers, the financial cost of acquiring foreign currency and losses when the exchange rate is unfavourable are some of the challenges that distributors have to face [13]. The income of distributors typically comes from payment by a regulated margin as a fixed percentage of the procured price. In some countries, a lower percentage is applied to higher priced stocks, thus resulting in a regressive margin. In some markets with a regulated or fixed margin, manufacturers may provide discounts while others may be prohibited from doing so, depending on their policies. A discount may be given when wholesalers can influence the sale of a product, especially when the product is a generic drug. The act of giving a discount has transformed into a “fee-for-service” model in some countries to encourage marginal negotiation between manufacturers and distributors [13].

While a wholesaler’s primary objective is to meet the market demand, which is often unpredictable and ever-changing, a wholesaler also has to assist retailers to prevent them from holding excessive inventory. Furthermore, a wholesaler aims to provide sufficient working capital for retailers to ensure they have the capability to purchase the stocks before receiving payment from end-users. To meet the objectives above, some wholesalers provide a series of commercial supports to help stabilize pharmacies’ businesses. The assistive supports include retailing management, sales training, accounting management and relevant business-related training [13].

## 7. Conclusions

In this review, the interplay of medicine pricing and the supply chain including mechanism and pharmaceutical price components and pharmaceutical supply chain were elucidated. Major components of the pharmaceutical value chain including manufacturers, market warehouses, distribution centres, wholesalers, and retailers were presented. Legislative context aspects such as regulation related to private healthcare facilities and services, tariffs exemptions and external and international reference pricing were also discussed. Lastly, patient medicine spending aspects including out-of-pocket spending and out-of-pocket costs were also reviewed. The regulation of mark-ups in the absence of any price control strategy causes medicine prices to be lowered. The implementation of this policy is less complicated compared to the other available options because it only demands a handful of cost and supply chain-related information, together with enforcement details. This policy may, however, cause a negative effect on availability and access through the changing of prices. Furthermore, there would be a risk of higher prices if the development of the mark-up structure lacked transparency.
